# Current Evidence and Future Directions for Colchicine in the Prevention of Atherosclerotic Cardiovascular Disease

**DOI:** 10.1055/a-2724-4543

**Published:** 2025-11-07

**Authors:** Aimée M.H. van Zelm, Aernoud T.L. Fiolet, Robert J. Hinchliffe, Shirley Jansen, Martin Teraa, Noel C. Chan

**Affiliations:** 1 Department of Vascular Surgery, University Medical Center Utrecht, Utrecht, The Netherlands; 2 Department of Cardiology, University Medical Center Utrecht, Utrecht, The Netherlands; 3 Department of Vascular Surgery, University of Bristol, Bristol, United Kingdom; 4 Department of Vascular and Endovascular Surgery, Sir Charles Gairdner Hospital, Perth, Western Australia, Australia; 5 Heart and Vascular Research Institute, Harry Perkins Institute of Medical Research, Perth, Western Australia, Australia; 6 Department of Medicine, McMaster University, Ontario, Canada

**Keywords:** colchicine, inflammation, cardiovascular disease, prevention, atherosclerosis

## Abstract

Chronic inflammation plays a key role in the development and progression of atherosclerotic cardiovascular disease (ASCVD) and its complications. Despite the use of blood pressure-, lipid-, and glucose-lowering therapies as well as antithrombotic agents, the lifetime residual cardiovascular (CV) risk in patients with ASCVD remains high. Because chronic inflammation remains an unaddressed risk factor, anti-inflammatory therapy has the potential to further lower residual CV risk in these patients. Low-dose colchicine (0.5 mg daily) has emerged as a promising low-cost oral anti-inflammatory therapy for this indication. In patients with chronic coronary syndrome (CCS), low-dose colchicine was well-tolerated and reduced the risk of myocardial infarction, stroke, coronary revascularization, and CV death. However, trials in patients with acute coronary syndrome (ACS) yielded conflicting results, and two trials in patients with ischemic stroke did not show a benefit. In patients with peripheral artery disease (PAD), preliminary observational data suggested a potential benefit, and a randomized trial is currently underway to examine its efficacy in reducing CV and limb events. The long-term safety data for low-dose colchicine in ASCVD are reassuring. Although pooled data from trials in ASCVD show a small (0.55%) absolute increase in the risk of hospitalization for gastrointestinal events, adverse signals were not observed for serious infection, cancer, or severe myotoxicity. In this article, we review the clinical studies of colchicine that examined its risk–benefit for the prevention of CV events in patients with ASCVD, discuss clinical and research implications, and highlight knowledge gaps.

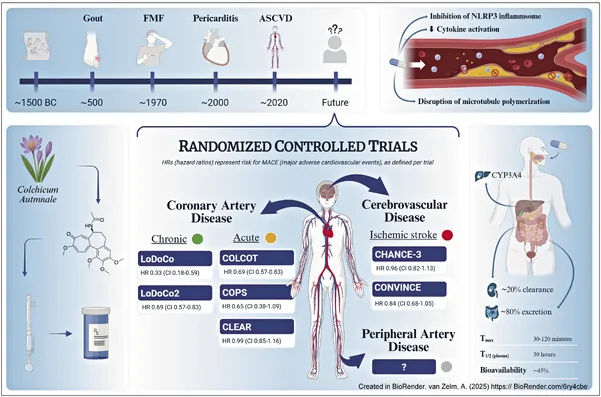

## Introduction

Atherosclerotic cardiovascular disease (ASCVD) remains a leading cause of morbidity and mortality worldwide, with notable disparities in risk factor management, health care access, and clinical outcomes between developed and developing countries. 1 2 The three main clinical presentations of ASCVD are coronary artery disease (CAD), cerebrovascular disease (CeVD), and peripheral artery disease (PAD).

Guideline-driven medical management for ASCVD includes (a) therapies that treat conventional risk factors (e.g., lipid-, blood pressure-, and glucose-lowering therapies); (b) antithrombotic therapies; and (c) behavioral interventions (e.g., smoking cessation, physical activity, and diet). Despite advances in these preventive therapies, ASCVD remains associated with a substantial risk of atherothrombotic complications, including major adverse cardiovascular (MACEs) or limb events (MALEs; see Table 1 ). 3 4 5 6 MACE refers to a composite outcome that includes non-fatal myocardial infarction (MI), non-fatal stroke, and cardiovascular (CV) death. MALE refers to a composite outcome that includes lower extremity ischemia requiring vascular intervention or major amputation. 7 8 Although residual risk persists in all ASCVD patients, the risk of MACE is twice as high in patients with PAD as compared to those with CAD. 3 6 9 Management of residual risk of future atherothrombotic complications in ASCVD patients necessitates patient-tailored treatments that target the strongest risk drivers, and novel therapeutic approaches that go beyond traditional risk factors.

**Table 1:** Real-world rates of mortality and major adverse events in atherosclerotic cardiovascular disease according to the SMART ( *n* = 9,005) and REACH ( *n* = 67,388) registries a

		Events per 100-person years		
		CAD	CeVD	Symptomatic PAD
All-cause mortality		1.8	2.7	3.8
MACE	Cardiovascular death	0.7–1.9	1.2–2.1	1.8–2.9
	Non-fatal myocardial infarction	0.9–1.2	0.4–0.9	0.8–1.2
	Non-fatal stroke	0.4–1.2	0.9–2.8	0.5–1.6
MALE		0.5	0.5	4.7

Abbreviations: CAD, coronary artery disease; CeVD, cerebrovascular disease; MACE, major adverse cardiovascular event; MALE, major adverse limb event; PAD, peripheral artery disease.

a Based on data from the REduction of Atherothrombosis for Continued Health Registry: An international, prospective, observational investigation in subjects at risk for atherothrombotic events (REACH registry) and the Utrecht Cardiovascular Cohort–Second Manifestations of Arterial Disease Study: Prospective cohort study of patients at high cardiovascular risk in the Netherlands (UCC-SMART). 3 4 5 6

Various processes contribute to residual atherothrombotic risk, which include lipid, thrombotic, metabolic, and inflammatory factors. 10 Of these, the inflammatory component remains mostly unaddressed in clinical practice, despite the widely recognized role of inflammation in atherosclerotic plaque formation, progression, and rupture. 11 Emerging data from clinical studies are supporting the paradigm that targeting inflammation could further reduce CV risk. 12 Colchicine is a widely available, low-cost, oral anti-inflammatory agent, and is being repurposed for this indication, with low dose (0.5 mg daily) showing promise in ASCVD. 13 14

This manuscript provides a comprehensive overview of colchicine, outlining its historical use and pharmacologic profile. Moreover, we review clinical studies in patients with ASCVD that have evaluated its risk–benefit profile for the prevention of CV events, discuss the clinical and research implications of the findings to date, and highlight the remaining knowledge gaps.

## Inflammation in Atherosclerosis

Inflammation plays a central role in the initiation, progression, and complications of atherosclerosis. 15 16 Accumulation of low-density lipoprotein cholesterol and its oxidized variants (oxLDL) in atherosclerotic plaques triggers a chronic inflammatory response. OxLDL induces the expression of adhesion molecules on the endothelial surface, facilitating the recruitment of various immune cells, including T lymphocytes and macrophages. 17 A key intracellular mediator of this inflammatory cascade is the nucleotide-binding domain, leucine-rich-containing family, pyrin domain-containing-3 (NLRP3) inflammasome, which becomes activated in response to oxLDL, cholesterol crystals, and other signals. Once activated, NLRP3 promotes caspase-1-dependent maturation of the proinflammatory cytokines interleukin (IL)-1β and IL-18, which amplify local inflammation, enhance immune cell recruitment, and contribute to plaque instability. These cytokines, along with IL-6 and tumor necrosis factor (TNF)-α, further exacerbate the process by promoting matrix degradation and thinning of the fibrous cap surrounding the necrotic lipid core. 17 Rupture or erosion of a vulnerable plaque releases the procoagulant contents of the plaque within the vessel lumen, resulting in clot formation and possible occlusion of the blood vessel (atherothrombosis). 18 Arterial occlusion results in end-organ ischemia and in the acute clinical complications of atherosclerosis, such as acute coronary syndrome (ACS), ischemic stroke, and acute limb ischemia. 19 Because inflammation is central to the initiation, progress, and complications of atherosclerotic disease, inflammatory pathways and mediators are attractive targets for developing novel therapeutic interventions. 20 21

Advances in the understanding of the inflammatory mechanisms of atherosclerosis have identified several promising targets for novel anti-inflammatory therapies. 22 Among these, the modulation of the NLRP3 inflammasome and interleukin pathway appears to be the most promising. 23 The pivotal Anti-inflammatory Therapy with Canakinumab for Atherosclerotic Disease (CANTOS) trial was the first to show that a monoclonal antibody (canakinumab) that neutralizes IL-1β, reduced CV events in patients with prior MI and elevated high-sensitivity C-reactive protein (hsCRP), without affecting lipid levels. Canakinumab (150 mg subcutaneously every 3 months) significantly reduced MACE (4.50 vs. 3.86 events per 100 person-years; number needed to treat approximately 156 per year), and this benefit was accompanied by a modest increase in fatal infection (0.28 vs. 0.18 events per 100 person-years; number needed to harm approximately 1,000 per year). Hence, CANTOS provided proof of concept of the anti-inflammatory paradigm in CVD. 24 Based on these considerations, targeting the NLRP3 inflammasome and interleukin pathway is a logical approach. By contrast, anti-inflammatory agents that do not effectively reduce inflammatory biomarkers in this pathway, such as IL-1β, IL-6, or hsCRP, have not been shown to reduce CV outcomes. For instance, the Cardiovascular Inflammation Reduction Trial (CIRT) evaluated low-dose methotrexate in patients with a history of MI or multivessel CAD, along with type 2 diabetes or metabolic syndrome. 25 Methotrexate did not reduce the rates of CV events, nor did it lower IL-1β, IL-6, or hsCRP levels. In contrast, canakinumab reduced IL-6 and hsCRP levels as well as CV events, but its high cost, subcutaneous administration, and the possible small increased risk of fatal infections limit its use. 24 The drug was not approved by regulators and has not been adopted in clinical practice for CV indications. 26 27 The majority of these novel anti-inflammatory compounds are also likely to be costly and inaccessible in resource-limited settings. In contrast, colchicine is a low-cost oral agent with broad anti-inflammatory effects, including the inhibition of the same inflammatory pathways. 28

## Colchicine: History, Pharmacology, and Clinical Use

Colchicine, originally derived from the autumn crocus ( *Colchicum autumnale* ), has a rich therapeutic history dating back to ancient Greece. 29 Colchicine was initially used for gout during the Byzantine period and later for familial Mediterranean fever in the 1970s. 30 31 32 Its clinical use expanded to pericarditis in the early 2000s. 33 Recently, low-dose colchicine has been repurposed for the prevention of atherothrombotic complications in ASCVD. 13

Colchicine has the chemical formula of C _22_ H _25_ NO _6_ and is identified by the International Union of Pure and Applied Chemistry (IUPAC) as N-[(7S)-1,2,3,10-Tetramethoxy-9-oxo-5,6,7,9-tetrahydrobenzo[a]heptalen-7-yl] acetamide. 34 Its anti-inflammatory effects are primarily mediated through microtubule polymerization inhibition, which disrupts cellular processes such as mitosis, intracellular transport, and cell migration. 35 More recent data indicate that colchicine also suppresses the release of proinflammatory cytokines (e.g., IL-1β, IL-6, IL-18) by inhibiting the NLRP3 inflammasome, and by inhibiting neutrophil activation and neutrophil–platelet interactions. 36 37 In addition, ex vivo studies show that low-dose colchicine significantly lowers levels of 37 inflammatory proteins, suggesting broad anti-inflammatory effects beyond NLRP3 inflammasome inhibition. 38 These mechanisms are thought to contribute to its atheroprotective properties.

After oral administration in healthy humans, colchicine is rapidly absorbed with an average bioavailability of 45% and peak plasma concentrations are reached within 30 to 120 minutes. 39 The drug is widely distributed, with a volume of distribution of 5 to 8 L/kg, and accumulates preferentially in leukocytes, where concentrations are higher than in plasma. 40 Colchicine has a plasma half-life of approximately 30 hours, with steady state concentrations reached after 5 to 7 days of daily dosing. 39 Colchicine is partially metabolized in the liver via cytochrome P450 3A4 (CYP3A4), with approximately 80% excreted unchanged in the feces and 10 to 30% cleared in the urine. 41 42 As a substrate of both CYP3A4 and P-glycoprotein (P-gp), the use of concomitant drugs that inhibit CYP3A4/P-gp should be avoided (e.g., clarithromycin, cyclosporine, azole antifungal). 43 Colchicine has minimal interactions with commonly used CV medications, including angiotensin-converting enzyme inhibitors, angiotensin II receptor blockers, anticoagulants, antiplatelets, antihypertensives, antidiabetics, and statins. 43 In ASCVD trials where over 90% of patients were receiving statin therapy, the incidence of rhabdomyolysis was very low; the trials show no or only a modest increase in reported myalgia. 44 45 46 Long-term use of colchicine has demonstrated a favorable safety profile in individuals with both CV and non-CV conditions. 47 48 The most common side effects of low-dose colchicine are mild gastrointestinal (GI) symptoms (e.g., diarrhea, nausea), which typically manifest early, are transient, and affect 5 to 15% of patients. 43 More severe GI symptoms requiring hospitalization are infrequent, and pooled data from trials in ASCVD show a small (2.1% vs. 1.5%) absolute increase in the risk of hospitalization for GI events when compared to control. 13 The maximum safe serum concentration of colchicine at a steady state is typically considered to be 3.0 μg/L, and pharmacokinetic modeling suggests that a daily colchicine dose of 0.5 mg does not result in serum levels exceeding this limit in healthy individuals, nor in patients with mild to moderate kidney impairment. 43 In some countries, the 0.5-mg colchicine formulation has more recently been introduced as a brand-name medication. This formulation remains significantly more expensive than the 0.6-mg formulation. As a result, the 0.6-mg formulation is sometimes used as a substitute in clinical practice, despite the lack of CV trial data supporting this dose in ASCVD. Such substitution is unlikely to adversely affect efficacy, because pharmacokinetic modelling data indicate minimal differences in plasma colchicine levels between the 0.5- and 0.6-mg doses in patients with normal renal function. 43 49 50 However, caution is required if this higher dose were to be used in patients with chronic kidney disease or in those receiving concomitant CYP3A4 or P-gp inhibitors.

Adverse events, such as severe bone marrow suppression, severe neuropathy, and rhabdomyolysis, are rare and associated with higher-than-recommended doses. 13 48 51 52 Overdose, typically involving ingestion of more than 0.5 to 0.8 mg/kg, can lead to severe complications, including multiorgan failure and death, underscoring the importance of adherence to recommended dosing. 52 53 54 Long-term use of low-dose colchicine has not been associated with increased risks of cancer, sepsis, cytopenia, myotoxicity, or changes in renal function. 43 55 56

## Colchicine in Atherosclerotic Cardiovascular Disease: Existing and Emerging Clinical Evidence

Several randomized controlled trials (RCTs) and observational studies have evaluated the role of colchicine in the prevention of atherothrombotic complications in ASCVD. In this section, we focus on the best available evidence, primarily derived from RCTs whenever such data exist. In cases where randomized trial data are lacking, we discuss exploratory findings from observational studies to provide the most comprehensive perspective possible.

A recent meta-analysis identified nine trials involving 30,659 patients with ASCVD, of whom 15,255 patients were randomized to colchicine and 15,404 to no colchicine or placebo, on top of usual care. 13 Most RCTs were conducted in patients with CAD, 44 45 57 58 59 and a few in CeVD. 46 60 To date, there is no completed RCT in PAD, but several observational studies examining its efficacy in the prevention of CV and limb events in PAD have been published. 61 62 63

### Coronary Artery Disease

Five randomized trials, that collectively included 18,656 patients, examined the efficacy and safety of low-dose colchicine in CAD: Two trials in 6,054 patients with CCS and three trials in 12,602 patients with ACS (see Table 2 ).

**Table 2:** Randomized controlled trials of low-dose colchicine in coronary artery disease

Study	Design	Population (sample size)	Median follow-up (study period)	Colchicine dosage	Primary outcome	HR (95% CI) *p* -value
LoDoCo 58	• Approximately 1:1 randomization • Open label • Blinded endpoint • Single-center	CCS ( *N* = 532)	36.0 months (August 2008–May 2012)	0.5 mg/day	Composite of ACS, OHCA, non-cardioembolic ischemic stroke	0.33 (0.18–0.59) *p* < 0.001
LoDoCo2 41	• 1:1 randomization • Placebo-controlled • Double-blind • Multicenter	CCS ( *N* = 5,522)	28.6 months (August 2014–February 2020)	0.5 mg/day	Composite of CV death, MI, ischemic stroke, ischemia-driven coronary revascularization	0.69 (0.57–0.83) *p* < 0.001
COLCOT 59	• 1:1 randomization • Placebo-controlled • Double-blind • Multicenter	MI <30 days ( *N* = 4,745)	22.6 months (December 2015–July 2019)	0.5 mg/day	Composite of CV death, resuscitated cardiac arrest, MI, stroke, urgent hospitalization for angina leading to coronary revascularization	0.77 (0.61–0.96) *p* = 0.02
COPS 45	• 1:1 randomization • Placebo-controlled • Double-blind • Multicenter	ACS ( *N* = 795)	12.2 months (December 2015–October 2019)	0–1 month: 1.0 mg/day 1–12 month: 0.5 mg/day	Composite of all-cause mortality, ACS, non-cardioembolic ischemic stroke, ischemia-driven urgent coronary revascularization	0.65 (0.38–1.09) *p* = 0.10
CLEAR-SYNERGY 57	• 1:1:1:1 randomization (2 × 2 factorial design; colchicine and spironolactone) • Placebo-controlled • Double-blind • Multicenter	PCI for ACS ( *N* = 7,062)	36.0 months (February 2018–August 2024)	February 2018–September 2020: • <70 kg = 0.5 mg/day • ≥70 kg = 1.0 mg/day	Composite of CV death, recurrent MI, stroke, ischemia-driven urgent coronary revascularization	0.99 (0.85–1.16) *p* = 0.93
	·			September 2020–August 2024: • 0.5 mg/day		

Abbreviations: ACS, acute coronary syndrome; CAD, coronary artery disease; CCS, chronic coronary syndrome; CI, confidence interval; COLCOT, COLchicine Cardiovascular Outcomes Trial; COPS, Colchicine in Patients with Acute Coronary Syndrome; CV, cardiovascular; HR, hazard ratio; LoDoCo, Low Dose Colchicine; LoDoCo2, Low Dose Colchicine for Secondary Prevention of Cardiovascular Disease; MI, myocardial infarction; OHCA, out-of-hospital cardiac arrest; PCI, percutaneous coronary intervention.

#### Trials in Chronic Coronary Syndrome

The Low-Dose Colchicine (LoDoCo) and Low Dose Colchicine for Secondary Prevention of Cardiovascular Disease (LoDoCo2) trials evaluated colchicine for the prevention of MACEs in CCS. The LoDoCo trial was a randomized, open-label trial with blinded endpoint adjudication. 58 LoDoCo randomized 532 patients in Australia with CCS to low-dose colchicine (0.5 mg daily) or no colchicine. Colchicine reduced the composite endpoint of ACS, out-of-hospital cardiac arrest, or non-cardioembolic ischemic stroke by 67% (hazard ratio [HR] 0.33, 95% confidence interval [CI] 0.18–0.59, *p* < 0.001) after a median follow-up of 36 months. Despite certain design limitations, this pioneering study provided the first proof of concept that long-term anti-inflammatory therapy with low-dose colchicine can reduce MACE in patients with CCS.

The LoDoCo2 trial was an international, double-blind, placebo-controlled randomized trial. 41 The study randomized 2,762 patients with CCS to colchicine 0.5 mg once daily and 2,760 patients to placebo. All participants first underwent a 30-day run-in period with colchicine 0.5 mg daily prior to randomization. In LoDoCo2, 65.5% of patients were included in the Netherlands and 34.5% in Australia. Colchicine reduced the risk of CV death, spontaneous MI, ischemic stroke, or ischemia-driven coronary revascularization by 31% (HR 0.69, 95% CI 0.57–0.83, *p* < 0.001) at a median follow-up of 28.6 months. 44 A reduction of CV events was observed across all prespecified subgroups. 64 Moreover, colchicine was shown to reduce inflammatory proteins both in the NLRP3- and non-NLRP3 pathways, as well as to stabilize atherosclerotic lesions. 38 For instance, an increase in dense calcified plaque volume was observed with colchicine therapy, which reflects a stabilizing effect on atherosclerotic lesions. 65

#### Trials in Acute Coronary Syndrome

The COLchicine Cardiovascular Outcomes Trial (COLCOT) was the first international randomized placebo-controlled trial to examine the efficacy and safety of low-dose colchicine in 4,745 patients with recent MI, who were randomly assigned to receive either 0.5 mg colchicine or placebo. 59 COLCOT enrolled participants across multiple countries, including Canada, South America, and Europe, although the proportion per region was not reported. The mean time of enrolment after MI was 13.5 days. Treatment with colchicine led to a 23% reduction in the composite endpoint of CV death, resuscitated cardiac arrest, MI, stroke, or urgent hospitalization for angina requiring coronary revascularization (HR 0.77, 95% CI 0.61–0.96, *p* = 0.02) after a median follow-up of 22.6 months. The benefit was primarily driven by reductions in urgent hospitalizations for angina leading to coronary revascularization (HR 0.50, 95% CI 0.31–0.81) and stroke (HR 0.26, 95% CI 0.10–0.70). A post hoc analysis revealed a more pronounced treatment benefit in patients who started colchicine within 72 hours after their event, compared to those who began treatment later. 66 There was no significant increase in serious adverse events (including serious GI events, fatal infection, septic shock, and cancer).

The Colchicine in Patients with Acute Coronary Syndrome (COPS) trial was a smaller multicenter, randomized, placebo-controlled trial. 45 COPS included 795 patients in Australia with ACS and evidence of CAD on coronary angiography. Approximately half of the patients were diagnosed with ST-elevation myocardial infarction (STEMI), while the other half had non-ST-segment elevation myocardial infarction (NSTEMI). Only a small proportion presented with unstable angina. Colchicine was initiated during hospitalization with a loading dose of 0.5 mg twice daily for 1 month, followed by a maintenance dose of 0.5 mg daily for 11 months. No significant difference was observed in the composite endpoint of all-cause mortality, ACS, ischemia-driven urgent revascularization, and non-cardioembolic ischemic stroke after 1 year (HR 0.65, 95% CI 0.38–1.09, *p* = 0.10). Concerns were raised about the significantly higher incidence of non-CV mortality in the colchicine group (five vs. zero, *p* = 0.023); however, the number of events was small. Although the primary cause of death was sepsis-related, a direct association with colchicine is unlikely, as three of the affected patients had discontinued the drug early in the trial. Despite the initial neutral results, an exploratory post hoc analysis suggested that colchicine might have a positive effect on urgent revascularization rates over time. A statistically significant reduction in the primary outcome was observed in patients receiving over 400 days of treatment (HR 0.47, 95% CI 0.27–0.82, *p* = 0.008). 45 This benefit remained consistent in an additional analysis encompassing 96% of study participants after 24 months of follow-up. 67

The Colchicine in Acute Myocardial Infarction (CLEAR-SYNERGY OASIS 9) trial is currently the largest, multicenter, randomized, placebo-controlled trial. 57 The trial used a 2 × 2 factorial design to examine the effect of low-dose colchicine versus placebo and spironolactone versus placebo. The study included 7,062 patients with MI who were candidates for percutaneous coronary intervention (PCI). Patients were recruited globally, with the largest contributions from North Macedonia (36.7%) and Canada (26.3%). Almost all patients were diagnosed with STEMI at presentation (95.1%), with a median time between diagnosis and enrolment of 26.8 hours. Colchicine dosing was weight-based at the beginning of the trial, with patients weighing ≥70 kg receiving 0.5 mg twice daily and those <70 kg receiving 0.5 mg once daily. After 90 days, all patients received 0.5 mg once daily. Following a blinded interim analysis, the initial weight-based dosing was replaced in the protocol with a fixed dose of 0.5 mg once daily for all patients, due to unexpectedly high rates of treatment discontinuation and the positive results from earlier trials using once daily dosing. There was no difference between the incidence of the primary efficacy outcome—a composite of CV death, MI, stroke, or ischemia-driven revascularization—between the colchicine and placebo arms (HR 0.99, 95% CI 0.85–1.16, *p* = 0.93) after a median follow-up of 3 years. No significant benefit was observed for any individual component of this composite outcome. Findings were consistent both within prespecified subgroups and in the on-treatment analysis. There was no significant difference in serious adverse events, but as expected, colchicine was associated with an increase in diarrhea. Although C-reactive protein (CRP) was not measured routinely, data from a subset of 2,803 patients demonstrated that colchicine significantly reduced CRP levels compared to placebo at 3 months, providing evidence of a measurable anti-inflammatory effect. However, in contrast to the CANTOS trial, data on treatment effect stratified by an inflammatory biomarker response are currently unavailable. 68

While there is no well-accepted explanation for the lack of colchicine's effect in CLEAR-SYNERGY when compared with COLCOT, several potential factors have been proposed. These include (a) differences in study populations, their risk profiles, and drug discontinuations, (b) differences in study design, including the unique challenges of conducting trials during the COVID-19 pandemic, and (c) random variation in results. Although both CLEAR SYNERGY and COLCOT included patients after MI, there were differences. For example, patients in CLEAR SYNERGY were randomized to colchicine earlier (median time 26.8 hours vs. 13.5 days), and there was a higher proportion of smokers (40.8% vs. 29.8%). However, despite the higher risk profile, CLEAR SYNERGY had a lower MACE rate in the control arm than in COLCOT (3.67 vs. 5.04 per 100 patient-years). While CLEAR SYNERGY had a higher cumulative rate of study drug discontinuation compared to COLCOT (26% vs. 18%), the on-treatment analysis still demonstrated no benefit. In addition, the conduct of CLEAR SYNERGY overlapped with the COVID-19 pandemic, with >70% of patients recruited during or after the pandemic. Others have described challenges in conducting trials during COVID-19 regarding study adherence, follow-up, and potential underreporting, as well as misclassification of CV events, which are supported by findings from epidemiological studies reporting reductions in hospitalizations and in MI during the COVID-19 shutdown. 69 70 71 Nonetheless, CLEAR SYNERGY was stopped after the prespecified number of primary outcome events was achieved, and outcome events underwent clinical adjudication, which would reduce the risk of misclassification.

Ongoing studies will help determine the magnitude and clinical relevance of colchicine's potential benefit in patients with CAD (see Table 3 ). 72 73 74 75 76 77 78 Notably, two upcoming trials will also specifically evaluate colchicine in patients undergoing PCI. In total, current ongoing studies are expected to enroll over 20,000 patients with CAD, covering a range of clinical settings.

**Table 3:** Ongoing randomized controlled trials of colchicine on the effect of mortality, major adverse cardiovascular event, or major adverse limb event in atherosclerotic cardiovascular disease a

Study, registry number (country of PI)	Design	Population (anticipated sample size)	Estimated follow-up duration (completion year)	Colchicine dosage	Primary outcome
ASCVD					
EPOCA, NCT06930885 (Brazil) 72	• 1:1:1:1 randomization (2 × 2 factorial design; colchicine and polypill) • Placebo-controlled • Double-blind • Multicenter	Previous ACS/ischemic stroke/acute limb ischemia/non-traumatic limb amputation/arterial revascularization/≥ 50% arterial obstruction ( *N* = 7,713)	36 months (2031)	0.5 mg/day	Composite of CV death, non-fatal MI, non-fatal stroke, urgent revascularization, non-traumatic major lower limb amputation
CAD					
COLCARDIO-ACS, ACTRN12616000400460 (Australia) 73	• 1:1 randomization • Placebo-controlled • Double-blind • Multicenter	ACS <30–45 days + hsCRP ≥2 mg/L ( *N* = 3,000)	36 months (2025)	0.5 mg/day	Composite of CV death, ACS, urgent revascularization, non-fatal stroke
RIGHT, NCT06025071 (China) 74	• 1:1 randomization • Open label • Blinded endpoint • Single center	Multivessel CAD + 60–80 years + hsCRP ≥2 mg/L ( *N* = 800)	12 months (2025)	0.5 mg/day	Composite of CV death, spontaneous (nonprocedural) MI, ischemia-driven coronary revascularization, ischemic stroke
COLT-HF, NCT05873881 (Canada) 75	• 1:1:1:1 randomization (2 × 2 factorial design; colchicine and thiamine) • Placebo-controlled • Double-blind • Multicenter	Ischemic HF ( *N* = 2,500)	42 months (2027)	0.5 mg/day	Composite of CV death, HF event, or CV event (MI/ ischemic stroke/ revascularization)
COL BE PCI, NCT06095765 (Belgium) 76	• 1:1 randomization • Placebo-controlled • Double-blind • Multicenter	PCI for ACS/CCS ( *N* = 2,770)	44 months (2028)	0.5 mg/day	Composite of all-cause mortality, non-fatal MI, non-fatal stroke, coronary revascularization
Efficacy and Safety of Colchicine After PCI, NCT06472908 (China) 77	• 1:1:1 randomization • Placebo-controlled • Double-blind • Multicenter	PCI for ACS/CCS ( *N* = 8,862)	24–48 months (2028)	1. 0.5 mg/day 2. 0.375 mg/day	Composite of CV death, non-fatal MI, non-fatal stroke, ischemia-driven coronary revascularization
TACTIC, NCT06215989 (China) 78	• 1:1 randomization • Placebo-controlled • Double-blind • Multicenter	ACS ( *N* = 6,574)	12 months (2028)	0.5 mg/day	Composite of CV death, non-fatal ischemic stroke, non-fatal spontaneous MI, readmission for ACS, ischemia driven (unplanned) revascularization
**CeVD**					
ARCHIMEDES, NCT06396858 (Brazil) 80	• 1:1:1:1 randomization (2 × 2 factorial design; colchicine and rivaroxaban) • Placebo-controlled • Double-blind • Multicenter	Ischemic stroke <14 days ( *N* = 4,500)	12 months (2026)	0.5 mg/day	1. Major bleeding 2. Composite of CV death, stroke, MI, urgent revascularization
RIISC-THETIS, NCT05476991 (France) 81	• 1:1:1:1 randomization (2 × 2 factorial design; colchicine and ticagrelor) • Open label • Multicenter	Ischemic stroke/TIA lasting >10 minutes + carotid/cerebral artery stenosis ( *N* = 2,800)	36 months (2027)	0.5 mg/day	1. Nonfatal ischemic stroke 2. Undetermined stroke 3. Nonfatal MI 4. Urgent coronary or carotid revascularization 5. CV death
CASPER, ACTRN12621001408875 (Australia) 82	• 1:1 randomization • Placebo-controlled • Double-blind • Multicenter	Ischemic stroke without major disability/TIA with imaging evidence + hsCRP ≥1 mg/L ( *N* = 1,500)	36 months (2027)	0.5 mg/day	Composite of non-fatal stroke, ACS, MI, urgent revascularization, CV death
CHANCE-3 EX, NCT07035405 (China) 83	• 1:1 randomization • Placebo-controlled • Double-blind • Multicenter	Minor–moderate ischemic stroke ( *N* = 7,500)	24 months (2028)	0.5 mg/day	Composite of ischemic stroke, MI, and CV death
PAD					
LEADER-PAD, NCT04774159 (Canada) 84	• 1:1 randomization • Placebo-controlled • Double-blind • Multicenter	Symptomatic lower extremity PAD ( *N* = 6,150)	36–60 months (2029)	0.5 mg/day	Composite of CV death, MI, stroke, severe limb ischemia requiring vascular intervention or major vascular amputation

Abbreviations: ACS, acute coronary syndrome; ASCVD, atherosclerotic cardiovascular disease; CAD, coronary artery disease; CCS, chronic coronary syndrome; CeVD, cerebrovascular disease; CHD, coronary heart disease; CV, cardiovascular; HF, heart failure; hsCRP, high-sensitivity C-reactive protein; MACE, major adverse cardiovascular event; MALE, major adverse limb event; MI, myocardial infarction; PAD, peripheral artery disease; PCI, percutaneous coronary intervention; PI, principal investigator; SOC, standard of care; TIA, transient ischemic attack.

a Randomized controlled trials registered on ClinicalTrials.gov/ClinicalTrialRegister.eu/EUClinicalTrials.eu/ANZCTR.org, with an anticipated enrollment of >500 participants and reporting MACE/MALE/mortality as a primary outcome (accessed July 8, 2025).

### Cerebrovascular Disease

The potential role of colchicine in CeVD has garnered attention following its efficacy in reducing ischemic strokes in the earlier trials in CAD. 79 Two trials have examined the efficacy of short- and long-term low-dose colchicine, respectively, in patients with ischemic stroke or high-risk transient ischemic attacks (TIAs; see Table 4 ).

**Table 4:** Randomized controlled trials of low-dose colchicine in cerebrovascular disease

Study	Design	Population (sample size)	Follow-up (study period)	Colchicine dosage	Primary outcome	HR (95% CI) *p* -value
CHANCE-3 60	• 1:1 randomization • Placebo-controlled • Double-blind • Multicenter	Minor–moderate ischemic stroke/TIA + hsCRP ≥2 mg/L ( *N* = 8,343)	3.0 months (August 2022–April 2024)	1–3 days: 1.0 mg/day 3–90 days: 0.5 mg/day	New stroke (ischemic of hemorrhagic) <90 days	0.98 (0.83–1.16) *p* = 0.79
CONVINCE 46	• 1:1 randomization • Open label • Blinded endpoint • Multicenter	Non-severe ischemic stroke/high-risk TIA ( *N* = 3,144)	33.6 months (December 2016–January 2024)	0.5 mg/day	Composite of first fatal or non-fatal recurrent ischemic stroke, MI, cardiac arrest, hospitalization for UA	0.84 (0.68–1.05) *p* = 0.12

Abbreviations: CHANCE, Colchicine in patients with acute ischaemic stroke or transient ischaemic attack; CI, confidence interval; CONVINCE, Long-term Colchicine for the Prevention of Vascular Recurrent Events in Non-Cardioembolic Stroke; HR, hazard ratio; hsCRP, high-sensitivity C-reactive protein; MI, myocardial infarction; TIA, transient ischemic attack; UA, unstable angina.

The Colchicine in patients with acute ischaemic stroke or transient ischaemic attack (CHANCE-3) trial was a multicenter, double-blind, randomized, placebo-controlled trial, conducted in China. 60 This short-term trial randomized 8,343 patients to low-dose colchicine or placebo. Patients were 40 years or older and had either an acute minor-to-moderate ischemic stroke or a high-risk TIA with evidence of vascular inflammation, as indicated by a baseline hsCRP level of ≥2 mg/L. Patients initiated trial medication within 24 hours of symptom onset and continued treatment for 90 days. There was no difference between the occurrence of the primary composite endpoint composed of recurrent stroke or other vascular events (HR 0.98, 95% CI 0.83–1.16, *p* = 0.79).

The Long-term Colchicine for the Prevention of Vascular Recurrent Events in Non-Cardioembolic Stroke (CONVINCE) trial was an international, randomized, open-label trial, with blinded endpoint adjudication. 46 The trial enrolled 3,144 patients aged 40 years or older, with non-severe ischemic stroke or high-risk TIA. The majority of patients were enrolled in the United Kingdom (38%), followed by Ireland (15%) and Germany (14%). Inclusion criteria required the qualifying event to be caused by large artery atherosclerosis, lacunar disease, or cryptogenic embolism, assessed by treating clinicians, with imaging evidence supporting the diagnosis. The qualifying event had to occur within 72 hours to 28 days before randomization. Participants were allocated to low-dose colchicine in addition to standard of care, or standard of care alone, for a median follow-up period of 33.6 months. In the primary intention-to-treat analysis, the primary composite outcome of non-fatal ischemic stroke, MI, cardiac arrest, hospitalization for UA, or vascular death was not significantly reduced (HR 0.84, 95% CI 0.68–1.05, *p* = 0.12), but the on-treatment analysis showed a significant reduction (HR 0.796, 95% CI 0.63–0.9992, *p* = 0.0492).

Despite differences in study design, populations, and treatment duration, both CHANCE-3 and CONVINCE did not show a reduction in ischemic stroke and CV events with low-dose colchicine. CHANCE-3 examined the short-term efficacy of colchicine and included patients with elevated inflammation soon after acute cerebrovascular events, whereas CONVINCE was a longer-term trial including patients up to 28 days after a cerebrovascular event. The treatment duration in CHANCE-3 may have been too short to detect a potential benefit, an effect that could have been observable in CONVINCE with its longer follow-up. However, CONVINCE was stopped prematurely before reaching the prespecified number of outcome events, potentially limiting its power to demonstrate efficacy. In addition, a lower-than-expected event rate, as well as a high study drug discontinuation rate, could have contributed to a reduced power to detect a difference, which was confirmed in the prespecified on-treatment analysis.

Several ongoing trials are evaluating colchicine in patients with CeVD (see Table 3 ). 80 81 82 83 Of particular interest, CHANCE-3 EX will include a longer treatment and follow-up period than CHANCE-3, which may provide additional insight into the long-term efficacy and safety of colchicine in this population.

### Peripheral Artery Disease

Currently, there are no published RCTs investigating the effects of colchicine in PAD. In addition, previous trials of colchicine in ASCVD have not reported PAD subgroup analyses, and MALE outcomes were not reported consistently. Current evidence is restricted to observational studies with inherent limitations. Three large observational studies have examined the efficacy of colchicine in PAD by comparing colchicine users with non-users in patients with gout and concomitant PAD (see Table 5 ).

**Table 5:** Observational studies of colchicine in peripheral artery disease

Study (year) Source	Design	Population (sample size)	Exposed (#1)	Control (#2)	Confounding adjustment	Follow-up duration and Outcome	Effect (cummulative incidence #1 vs. #2)
Heindel et al (2024) 61 US Medicare	Retrospective cohort study with target trial emulation	PAD and gout ( *N* = 1,820)	Colchicine	NSAID	Inverse probability weighting for 1. age 2. sex 3. race 4. comorbidities 5. general health 6. gout severity 7. PAD severity 8. medication history 9. calendar time 10. enrolment duration prior to treatment	2-year follow-up Composite of hospitalization for MI, acute stroke/TIA, coronary revascularization, above-ankle amputation, iliac/infrainguinal arterial revascularization	RR 0.95 95% CI 0.83–1.07 *p* = NR (29.9% vs. 31.5%)
			Long-term colchicine (>3 months)	Short-term colchicine (<3 months)			RR 0.92 95% CI 0.70–1.16 *p* = NR (30.7% vs. 33.4%)
Lin et al (2024) 62 Taiwan NHIRD	Retrospective cohort study	PAD ( *N* = 60,219 pairs)	Colchicine	No colchicine	Propensity score 1:1 matching, for 1. age 2. sex 3. comorbidities 4. history of events 5. PAD severity 6. medication history 7. follow-up years	4.5-year follow-up Composite of lower limb revascularization and nontraumatic major amputation	SHR 0.75 95% CI 0.71–0.80 *p* < 0.001 (3.8% vs. 5.0%)
						4.5-year follow-up Composite of CV death, MI, ischemic stroke, coronary revascularization	SHR 0.84 95% CI 0.82–0.86 *p* < 0.001 (18.7% vs. 20.4%)
Tramujas et al (2024) 63 TriNetX network	Retrospective cohort study	Lower extremity PAD ( *N* = 52,350 pairs)	Colchicine	No colchicine	Propensity score 1:1 matching, for 1. age 2. sex 3. ethnicity 4. medication history 5. CV risk factors 6. rheumatic diseases	10-year follow-up Composite of all-cause mortality, MI, ischemic stroke, lower limb amputation, revascularization for lower limb ischemia	HR 0.90 95% CI 0.88–0.92 *p* < 0.001 (43.2% vs. 46.0%)

Abbreviations: CV, cardiovascular; HR, hazard ratio; MI, myocardial infarction; NR, not reported; NSAID, non-steroidal anti-inflammatory drug; PAD, peripheral artery disease; RR, relative risk; SHR, subdistribution hazard ratio; TIA, transient ischemic attack.

Heindel et al emulate two hypothetical randomized trials to estimate the real-world effectiveness of colchicine in patients with comorbid gout and PAD, using data from Medicare and a multicenter academic health system in the United States. 61 The study included 1,820 patients and compared colchicine initiators to non-steroidal anti-inflammatory drug initiators (emulated Trial 1), and long-term colchicine treatment to short-term treatment (emulated Trial 2). Over 2 years, no statistically significant difference in the composite outcome of MALE, MACE, and death was observed between the groups (Trial 1: RR 0.95, 95% CI 0.83–1.07; Trial 2: RR 0.92, 95% CI 0.70–1.16). The findings provided no conclusive evidence that colchicine reduced MACE, MALE, or death in this population, as the wide CIs could not exclude the possibility of a 20 to 30% risk reduction, which would be widely regarded as clinically relevant.

Lin et al analyzed the National Health Insurance Research Database (NHIRD), a claims database covering approximately 99% of the Taiwanese population. 62 This study included patients with gout and PAD, comparing colchicine users to non-users. About 60,219 patients were matched 1:1 using propensity score matching to mitigate confounding. Colchicine use was defined as receiving at least 90 days of colchicine prescriptions during the follow-up period of 4.5 years. Colchicine users had a significantly lower risk of MALE (HR 0.75, 95% CI 0.71–0.80, *p* < 0.001), including both lower limb amputations (HR 0.79, 95% CI 0.72–0.86, *p* < 0.001) and revascularization for limb ischemia (HR 0.76, 95% CI 0.71–0.80, *p* < 0.001). In the colchicine group, MACE rates were also lower (HR 0.84, 95% CI 0.82–0.86, *p* < 0.001), driven largely by a reduced risk of CV death (HR 0.75, 95% CI 0.73–0.78, *p* < 0.001). The study's definition of colchicine use (≥90 days) leaves uncertainty regarding long-term adherence or the duration of treatment.

Tramujas et al used the TriNetX network database, a global federated database of real-world clinical data from health care organizations across various countries. 63 After 1:1 propensity score matching colchicine users with non-users, 52,350 patients in each group were analyzed. Over a 10-year follow-up, colchicine use was associated with a reduced risk of both MACE (HR 0.93, 95% CI 0.91–0.95, *p* < 0.001), MALE (HR 0.80, 95% CI 0.82–0.88, *p* < 0.001), and all-cause mortality (HR 0.90, 95% CI 0.87–0.92, *p* < 0.001).

Findings from these observational studies are hypothesis-generating and underline the need for a well-designed RCT to provide definite efficacy and safety data for colchicine in the prevention of MACE and MALE in PAD. The ongoing Low Dose ColchicinE in pAtients With Peripheral Artery DiseasE to Address Residual Vascular Risk trial (LEADER-PAD, NCT04774159) is the first adequately powered multinational randomized placebo-controlled trial that is examining the risk–benefit of low-dose colchicine in the prevention of MACE and MALE in patients with symptomatic PAD (see Table 3 ). 84 The future findings of this trial could clarify colchicine's role in this high-risk population with ASCVD, in which inflammation and atherothrombosis burden are the highest. 14

## Implications for Clinical Practice and Future Directions

**Fig. 1:**
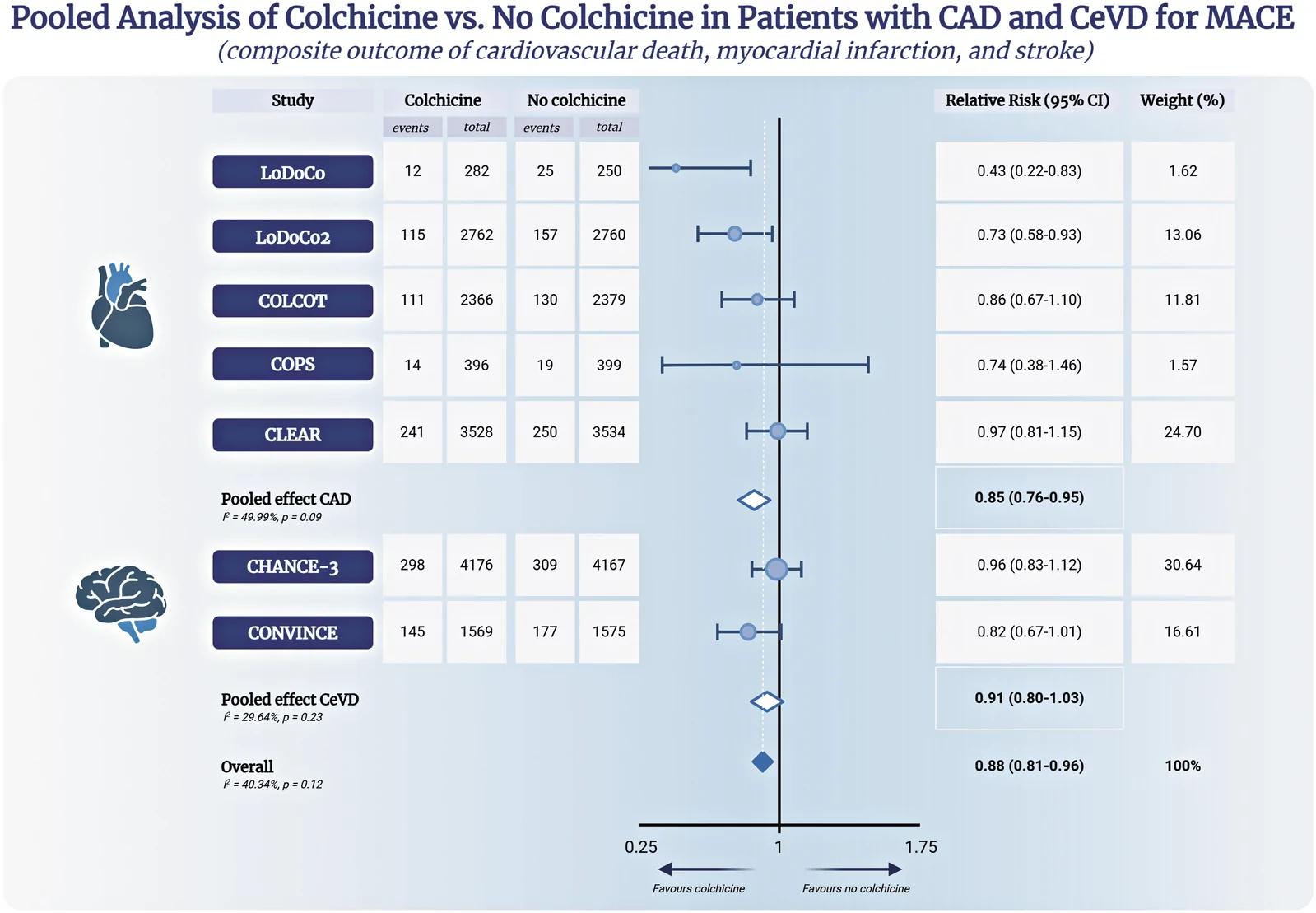
Pooled analysis of colchicine versus no colchicine in patients with CAD and CeVD for MACE (composite outcome of cardiovascular death, myocardial infarction, and stroke). Descriptive and outcome data from the principal and subsidiary publications were compiled for each trial, with additional data collected from collaborating investigators when possible. CAD, coronary artery disease; CeVD, cerebrovascular disease; CHANCE-3, Colchicine in patients with acute ischaemic stroke or transient ischaemic attack; CI, confidence interval; COLCOT, COLchicine Cardiovascular Outcomes Trial; CONVINCE, Long-term Colchicine for the Prevention of Vascular Recurrent Events in Non-Cardioembolic Stroke; COPS, Colchicine in Patients with Acute Coronary Syndrome; LoDoCo, MACE, major adverse cardiovascular event. Created in BioRender. van Zelm A. (2025). https://BioRender.com/5yltqij .

Based on earlier findings from LoDoCo2 and COLCOT, several guidelines have addressed the use of low-dose colchicine in patients with CAD on top of usual prevention strategies. 85 86 87 88 In the newly published ACC/AHA 2025 guideline for the management of ACS, colchicine is assigned a Class IIb recommendation (Level of Evidence C), indicating potential benefit in selected patients but highlighting uncertainty. 88 It is worth noting that colchicine is not currently included in guideline recommendations for PAD or secondary stroke prevention. 89 90 91 In some countries, such as the United States and Canada, low-dose colchicine has been approved for this indication by regulators. However, the adoption of low-dose colchicine for ASCVD prevention remains limited in clinical practice. Concerns about GI side effects, and the recent lack of significant results in CLEAR SYNERGY, CONVINCE and CHANCE-3 may in part contribute to poor adoption of this low-cost anti-inflammatory therapy. Reassuringly, in the latest meta-analysis involving over 15,000 patients receiving colchicine and a similar number receiving no colchicine or placebo, the totality of evidence to date continues to indicate that for ASCVD patients, (a) colchicine had a modest benefit, with a best estimate for relative risk (RR) reduction of 12% for MACE ( Fig. 1 presents the effect estimates stratified by ASCVD phenotype); (b) except for transient and mostly mild GI side effects, colchicine has not been associated with serious adverse complications; and (c) colchicine has not been associated with excess non-CV deaths. 13 This modest benefit is comparable in magnitude to that observed with other CV therapies, including the effect of aspirin found in large-scale meta-analyses involving 95,000 patients, and other lipid-lowering therapies like ezetimibe and PCSK9 inhibitors. 92 93 Furthermore, cost-effectiveness analyses demonstrate favorable ratios in studies evaluating the use of colchicine for the prevention of MACE in patients with CAD. 94 95 The current findings continue to support guideline recommendations, and adoption could be bolstered by the conduct of more definitive trials of colchicine in the various manifestations of ASCVD ( Table 3 ).

Current guidelines recommend a multidrug approach that targets distinct, but interrelated risk pathways involved in ASCVD (e.g., lipids, thrombosis, blood pressure, glucose control, inflammation). 85 88 89 91 As a result, patients are often prescribed multiple classes of medications for optimal prevention of CV complications but on the other hand, polypharmacy may have an adverse impact, including poor drug adherence. 96 97 Therefore, in patients for whom pill burden is a significant concern, selection among the available drug classes becomes a consideration. In the absence of a head-to-head comparison of various drug classes, selection is guided by a careful personalized risk–benefit assessment. This includes factors, such as the presence of elevated inflammatory markers, renal function, and the broader CV, renal, and metabolic benefits associated with specific agents, as well as shared decision-making with the patient.

The identification of patient subgroups most likely to benefit from colchicine remains a priority. Among inflammatory markers, hsCRP has emerged as a potentially useful tool for stratifying residual inflammatory and CV risk, but robust clinical evidence that supports a tailored approach to using colchicine based on hsCRP is lacking. Observational data suggest that individuals with hsCRP levels above 2 mg/L have higher CV risk and raise the possibility that they could derive greater benefit from anti-inflammatory therapies, and could guide physicians' decisions when faced with the issue of polypharmacy and the decision to add colchicine. 49 98 Patients with moderate to severe renal dysfunction (eGFR <30–50 mL/min/1.73 m ^2^ ) often exhibit elevated levels of inflammatory markers and have high CV risk, 99 but they were excluded from major ASCVD colchicine trials, which limits the generalizability of these findings to this population. This, in combination with colchicine's partial renal clearance, underscores the need for careful consideration when prescribing the drug in patients with chronic kidney disease, and further studies are needed to define its dosing, safety, and efficacy in this group. 100 Moreover, in patients for whom the residual risk is driven by metabolic features, such as those with type 2 diabetes, or in patients with chronic kidney disease, agents such as sodium/glucose cotransporter (SGLT) 2 inhibitors or glucagon-like peptide (GLP) 1 receptor agonists may offer additional organ-protective effects beyond atherosclerotic risk reduction and may therefore be preferred over colchicine. 100 101

Further research should focus on identifying which subgroups of patients with atherosclerosis derive the greatest benefit from low-dose colchicine. Subgroup analyses of existing trials and meta-analyses have so far not revealed consistent predictors of enhanced benefit. However, future analyses using patient-level data in meta-analyses, or biological substudies incorporating imaging and plasma biomarkers of inflammation, may provide deeper insights into individuals with high inflammatory risk who could benefit even more from colchicine. With several trials of colchicine ongoing, which are expected to cumulatively enroll another 35,000 patients in the next 5 years (see Table 3 ), we are likely to have a more precise estimate of treatment effects on various vascular outcomes and more definite answers on the efficacy of colchicine in various populations.

## Conclusion

In conclusion, the totality of data from randomized trials to date indicates that low-dose colchicine reduces MACE in patients with ASCVD and has a favorable safety profile. Although colchicine has been associated with GI side effects, it has not been associated with serious adverse events, such as serious infection, cancer, bone marrow suppression, neuropathy, myopathy, or excess non-CV mortality. If findings from ongoing trials in various manifestations of ASCVD and subpopulations are positive, the comparatively low cost of colchicine (vs. other novel anti-inflammatory molecules) may help promote its broader adoption in the future to reduce residual CV risk.

## Notes

**What is known about this topic?**

- Chronic inflammation plays a critical role in the development and progression of ASCVD, yet it remains an undertreated risk factor in clinical practice.
- Canakinumab, a monoclonal antibody inhibiting IL-1β, demonstrated that anti-inflammatory therapy can reduce CV risk in patients with MI, prompting the clinical evaluation of other anti-inflammatory drugs in ASCVD.
- Low-dose colchicine (0.5 mg daily) has emerged as a promising low-cost anti-inflammatory therapy to reduce the residual CV risk in patients with chronic CAD, but its efficacy remains unclear in other manifestations of ASCVD.

**What does this paper add?**

- Provides a comprehensive overview of the risk–benefit profile of low-dose colchicine across a broad range of ASCVD manifestations, based on latest data derived from recent studies.
- Discusses considerations on how to optimize the net clinical benefit of colchicine, and how to identify patient subgroups who may benefit most or be at increased risk of harm.
- Highlights key knowledge gaps, uncertainty in the evidence, as well as challenges in the clinical implementation of low-dose colchicine for the secondary prevention of CV complications in ASCVD.
- Provides an overview of ongoing and upcoming trials investigating colchicine in diverse ASCVD populations.
